# Methylphenidate Attenuates Limbic Brain Inhibition after Cocaine-Cues Exposure in Cocaine Abusers

**DOI:** 10.1371/journal.pone.0011509

**Published:** 2010-07-09

**Authors:** Nora D. Volkow, Gene-Jack Wang, Dardo Tomasi, Frank Telang, Joanna S. Fowler, Kith Pradhan, Millard Jayne, Jean Logan, Rita Z. Goldstein, Nelly Alia-Klein, Christopher Wong

**Affiliations:** 1 National Institute on Drug Abuse, Bethesda, Maryland, United States of America; 2 Laboratory of Neuroimaging, National Institute on Alcohol Abuse and Alcoholism, Bethesda, Maryland, United States of America; 3 Medical Department, Brookhaven National Laboratory, Upton, New York, United States of America; L'université Pierre et Marie Curie, France

## Abstract

Dopamine (phasic release) is implicated in conditioned responses. Imaging studies in cocaine abusers show decreases in striatal dopamine levels, which we hypothesize may enhance conditioned responses since tonic dopamine levels modulate phasic dopamine release. To test this we assessed the effects of increasing tonic dopamine levels (using oral methylphenidate) on brain activation induced by cocaine-cues in cocaine abusers. Brain metabolism (marker of brain function) was measured with PET and ^18^FDG in 24 active cocaine abusers tested four times; twice watching a Neutral video (nature scenes) and twice watching a Cocaine-cues video; each video was preceded once by placebo and once by methylphenidate (20 mg). The Cocaine-cues video increased craving to the same extent with placebo (68%) and with methylphenidate (64%). In contrast, SPM analysis of metabolic images revealed that differences between Neutral versus Cocaine-cues conditions were greater with placebo than methylphenidate; whereas with placebo the Cocaine-cues decreased metabolism (p<0.005) in left limbic regions (insula, orbitofrontal, accumbens) and right parahippocampus, with methylphenidate it only decreased in auditory and visual regions, which also occurred with placebo. Decreases in metabolism in these regions were not associated with craving; in contrast the voxel-wise SPM analysis identified significant correlations with craving in anterior orbitofrontal cortex (p<0.005), amygdala, striatum and middle insula (p<0.05). This suggests that methylphenidate's attenuation of brain reactivity to Cocaine-cues is distinct from that involved in craving. Cocaine-cues decreased metabolism in limbic regions (reflects activity over 30 minutes), which contrasts with activations reported by fMRI studies (reflects activity over 2–5 minutes) that may reflect long-lasting limbic inhibition following activation. Studies to evaluate the clinical significance of methylphenidate's blunting of cue-induced limbic inhibition may help identify potential benefits of this medication in cocaine addiction.

## Introduction

Dopamine (DA), a neurotransmitter that processes reward and prediction of reward [1], [2] is involved with the reinforcing effects of drugs of abuse [3], [4] and with responses to drug conditioned cues [5], [6], [7]. Specifically, fast and large DA increases, as those achieved with phasic DA cell firing (15–30 Hz), are implicated in both drug reward [8], [9] and conditioned cues [10], [11]. Indeed imaging studies have shown that the fast DA increases achieved with intravenous administration of methylphenidate (stimulant drug that increases DA by blocking DA transporters) are associated with its reinforcing effects [12] whereas similar but slower DA increases after oral administration of methylphenidate (MP) are not perceived as reinforcing [13]. Similarly the fast DA increases as triggered by conditioned cues [10], [11] are linked with craving responses in cocaine abusers [14], [15] whereas a similar magnitude of DA increases but achieved slowly (as induced by oral administration of MP) do not elicit craving [16]. Tonic DA cell firing (2–10 Hz), which results in stable and lower DA levels, appears to modulate such phasic DA cell firing via activation of inhibitory DA autoreceptors [17]. It has been hypothesized that repeated drug use results in increases in tonic DA levels that dampen phasic DA responses driving compulsive drug administration as an attempt to overcome the attenuated phasic DA signaling [18]. However, imaging studies performed in cocaine abusers studied during early or protracted detoxification have shown evidence of decreased striatal DA activity [19], including decreases in non-stimulated DA release [20], which suggest a decrease in tonic DA cell firing. Since low tonic DA levels would enhance phasic DA cell firing in response to conditioned-cues [21] we reasoned that increasing tonic DA levels in cocaine abusers would attenuate phasic DA responses to conditioned-cues and blunt the downstream activation of regions and circuits associated with these responses.

To test this hypothesis we assessed the effects of raising tonic DA levels by giving a low dose of oral MP (20 mg) [22] on the regional brain metabolic activation induced by cocaine-cues in cocaine abusers. We used Positron Emission Tomography (PET) and 2-deoxy-2[^18^F]fluoro-D-glucose (^18^FDG) to measure brain glucose metabolism (marker of brain function) [23] in 24 active cocaine abusers. Subjects were tested on four different days each under a different condition; twice while watching a neutral video (nature scenes), once with placebo and once with MP (20 mg po), and twice while watching a video of cocaine-cues (repeating scenes of people taking cocaine), once with placebo and once with MP. The cocaine-cues video used was one we had previously shown to induce significant increases in striatal DA, which was an effect associated with increases in drug craving in cocaine abusers [14]. We chose a dose of MP (20 mg oral), which we had previously shown induced significant increases in striatal DA in cocaine abusers but that did not elicit drug craving nor was it perceived as reinforcing [16]. Cocaine craving was measured using a brief version of the Cocaine Craving Questionnaire (CCQ) that evaluates current cocaine craving [24].

We hypothesized that MP by increasing tonic DA levels would dampen phasic DA firing triggered by conditioned cues and thus the activation pattern elicited by cocaine-cues would be attenuated when MP was given as compared to when placebo was given. We also hypothesized that this would be associated with reduced craving.

## Results

### Concentration of MP in plasma

Plasma MP levels did not differ between conditions and corresponded for the Neutral and the Cocaine-cues conditions respectively, at 30 minutes post administration to: 3.0±3 and 3.3±3 ng/ml; at 60 minutes to: 5.7±3 and 5.6±4 ng/ml; at 90 minutes to: 6.4±2 and 6.1±3 ng/ml; and at 120 minutes to: 6.1±2 and 5.7±2 ng/ml. Plasma MP levels were not significantly correlated with cardiovascular, behavioral or brain metabolic measures.

### Cardiovascular measures

Heart rate and systolic blood pressure (BP) increased significantly for all conditions (pre vs post) (Table 1). Comparisons between conditions were significant for heart rate (Repeated ANOVA F = 7, df 3, 69, p<0.0005) and systolic BP (Repeated ANOVA F = 4, df 3, 69, p<0.02) but not for diastolic BP (Table 1). For heart rate the comparisons between Neutral and Cocaine-cues were significant (P<0.05); the comparisons between Placebo and MP were significant (p<0.01) and the interaction (Drug by Video) did not reach significance (p<0.08). For systolic BP the comparisons between Neutral and Cocaine-cues were significant (p<0.01), the comparisons between Placebo and MP were significant (p<0.05) and the interaction (Drug by Video) was significant (p<0.02).

**Table 1:** Percent change in cardiovascular measures from the baseline state (prior to video and placebo exposures) to measures averaged during exposure to the videos (post).

	Neutral Placebo	Cocaine-cues Placebo	Neutral MP	Cocaine-cues MP	ANOVA
Heart Rate	6.5%±6	6.0%±6	8.2%±5	12.7%±8*	F = 7, p<0.0005
Systolic BP	2.5%±4	1.8%±6	1.4%±6	6.6%±6*τ	F = 4, p<0.02
Diastolic BP	−0.1%±5	−0.4%±6	0.6%±6	2.6%±7	F = 1.5, p<0.21

Measures correspond to mean and SD. ANOVA results correspond to the comparisons of the percent changes across conditions (df 3, 69).

*Connotes significant increase between the MP/Neutral and the MP/Cocaine-cues.

τ Significant interaction effect between video and drug condition.

### Craving measures

The craving measures (CCQ and self-reports) increased significantly with the Cocaine-cues exposure for both conditions (placebo or MP). For CCQ repeated ANOVA (measures prior to drug exposure, measures prior to video exposure, and measures post video exposure) differed significantly for the Cocaine-cues when given with PL (p<0.002) and when given with MP (p<0.003) but did not differ for the Neutral video conditions (PL or MP) (Table 2). The self-reports of craving were significantly higher for the Cocaine-cues video condition both for PL (p<0.002) and for MP (p<0.001). The interaction between drug and video condition was not significant.

**Table 2:** Scores on the measures of cocaine craving obtained with the CCQ and with self-reports of craving for the 4 conditions.

CCQ	Pre PL or MP	Pre Video	End video	ANOVA
Neutral PL	29±11	27±11	27±12	F = 0.4 p<0.64
Neutral MP	29±10	30±11	29±11	F = 1, p<0.36
Cocaine-cues PL	31±12	30±11	35±14	F = 5, p<0.008
Cocaine-cues MP	30±12	29±10	35±13	F = 7, p<0.002
Self-reports				
Neutral PL	2.7±2	2.3±2	2.2±1	F = 1, p<0.32
Neutral MP	2.7±2	2.4±2	2.7±2	F = 0.4, p<0.70
Cocaine-cues PL	2.9±3	2.5±2	4.2±3	F = 7, p<0.002
Cocaine-cues MP	3.0±3	2.8±2	4.6±3	F = 8, p<0.001

ANOVA results correspond to the comparisons within conditions (df 2, 46). The interaction between drug (placebo vs methylphenidate) by video condition (Neutral vs Cocaine-cues) was not significant. PL =  placebo, MP  =  methylphenidate.

### Absolute metabolic measures

Whole brain absolute metabolic measures corresponded for Neutral with PL to 34.9±5 µmol/100 g/min, for Neutral with MP to 36.1±5 µmol/100 g/min, for Cocaine-cues with PL to 35.0±5 µmol/100 g/min and for Cocaine-cues with MP to 35.9±5 µmol/100 g/min and the measures did not differ between conditions. The SPM analysis also showed no differences between conditions for the absolute metabolic images.

### Normalized metabolic measures

**Figure 1:**
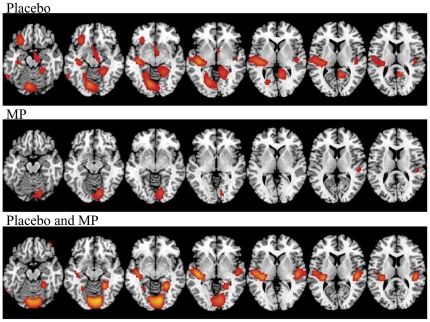
SPM results on the Video comparison (Neutral vs Cocaine-cues video) for the Placebo (PL), for the Methylphenidate (MP) and for both conditions (PL and MP) done on normalized metabolic images (Cocaine-Cues < Neutral). The Cocaine-cues lowered glucose metabolism and this effect was attenuated when subjects were given MP. Threshold of significance p<0.005 uncorrected, cluster size >200 voxels.

SPM comparisons for the normalized metabolic measures showed significant decreases in relative metabolism for the Cocaine-cues video when compared with the Neutral-video that were more extensive with Placebo than with MP. When Placebo was given decreases were significant in left limbic regions (lateral orbitofrontal cortex (OFC)/anterior insula (BA 47 and BA 11), accumbens, posterior insula) and right parahippocampal gyrus (BA 36 and 37), and in auditory (left BA 22 and left and right BA 42) and visual areas (left lingual gyrus) (Figure 1 and Table 3 for location of areas). In contrast when MP was given the only significant differences were in the right visual (inferior BA 18) and right auditory (BA 42) regions (Figure 1, Table 3 shows location of areas).

**Table 3:** SPM results for the comparison between the Cocaine-cues and the Neutral videos (Cocaine-cues < Neutral) when given with placebo and when given with methylphenidate (p<0.005 uncorrected, cluster size >200 voxels).

Cocaine-cues vs Neutral Placebo	Cluster size (# voxels)	T and p	x, y, z
L Lingual gyrus	1131	3.86 p<0.001	−16 −68 −6
L Posterior insula	1986	3.84 p<0.001	−40 −22 −4
L BA 47/11-anterior insula	614	4.42 p<0.001	−26 26 −14
R Parahippocampal	1183	4.20 p<0.001	24 −46 −10
L NAc	486	3.65 p<0.001	−2 14 −10
R BA 41/42	223	3.54 p<0.001	46 −22 10
**Cocaine-cues vs Neutral Methylphenidate**			
R Lingual gyrus	790	3.61 p<0.001	14 −80 −10
R BA 41/42	687	3.25 p<0.001	50 −20 10

Table shows, cluster size, T value of the comparison, significance (p) and location of coordinates (in mm) on Montreal Neurological Institute (MNI) coordinates (x,y,z). L  =  left, R  =  right.

**Figure 2:**
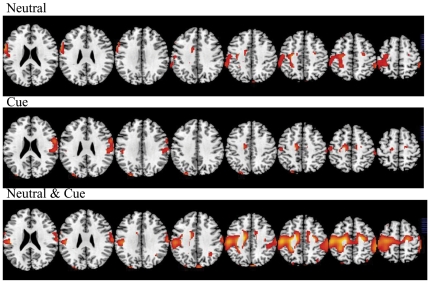
SPM results on the Drug comparison (Placebo vs MP) for the Neutral Video, for the Cocaine-Cues Video and for both conditions (Neutral and Cocaine-Cues) done on normalized metabolic images (MP < PL). MP decreased metabolism in superior parietal regions. Threshold of significance p<0.01 uncorrected, cluster size >200 voxels.

To control for potential findings driven by the effects of MP in the Neutral video we also compared the effects of MP with those of Placebo. The SPM analysis revealed significant decreases for both video conditions in precentral gyrus (BA 6) and with the Neutral video additional decreases in posterior cingulate (BA 30), superior temporal and postcentral gyrus (Figure 2, Table 4).

**Table 4:** SPM results for the comparison between Methylphenidate (MP) and Placebo (MP < Placebo) for the Neutral and the Cocaine-cues conditions (p<0.01 uncorrected, cluster size >200 voxels).

MP vs Placebo Neutral	Cluster size (# voxels)	Z-score	p values	x, y, z
Posterior Cingulate BA 30	1142	3.9	p<.001	8, −52, 8
Superior Temporal BA 42	503	3.9	p< 001	−66, −22, 10
Uncus BA 28	758	3.4	p<.002	30, 4, −38
Precentral gyrus BA 6	282	3.3	P<.002	−62, 4, 24
Postcentral gyrus BA 3	145	3.2	p<.002	−28, −16, 48
**MP vs Placebo Cocaine-cues**				
Precentral gyrus BA 6	175	3.1	p<.002	60, 2, 24

Table shows, the regions, cluster size, T value of the comparison, significance (p) and location of coordinates (in mm) on Montreal Neurological Institute (MNI) coordinates (x,y,z). L  =  left, R  =  right.

**Figure 3:**
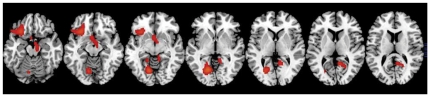
SPM results for the Video (Neutral vs Cocaine-cues) by Drug (Placebo vs MP) interaction (MP < PL). Methylphenidate resulted in less metabolic changes than the Placebo and there were no regions were the differences were greater for Methylphenidate than for the Placebo conditions. Threshold of significance p<0.01 uncorrected, cluster size >200 voxels.

The SPM analysis of the interaction effect between video and drug condition showed that the decreases with the Cocaine-cues were attenuated when MP was given when compared to placebo. Specifically the decreases when the cocaine-cues were given with placebo versus when given with MP were significantly greater in the right and left lingual gyrus, left hippocampal gyrus, left accumbens/BA 25 region and left lateral OFC (BA 47) (Figure 3, and Table 5 for location of areas).

**Table 5:** SPM results for the Video (Neutral vs Cocaine-cues) by Drug (Placebo vs MP) Interaction (MP < PL).

Region	Cluster # voxels	T, *p*	x,y,z
R Lingual Gyrus	788	3.95, *p<0.0001*	10, −50, 6
L Lingual Gyrus L Hippocampal Gyrus	1082	3.79, *p<0.001*	−12, −72, −10−18, −44, −6
L NAc/BA 25	287	3.29, *p<0.002*	−4,10, −8
L BA 47	407	3.12, *p<0.002*	−26, 24, −12

Table shows the regions, cluster size, T value, significance (p) and location of coordinates (in mm) on Montreal Neurological Institute (MNI) coordinates (x,y,z). When given MP the differences between the Neutral vs the Cocaine-cues video was less than when given placebo. L  =  left, R  =  right.

### Correlations between normalized metabolic changes and craving

Correlation analysis between the regions where SPM showed significant differences between the Neutral video and the Cocaine-cues video and self-reports of craving (delta scores pre vs post video exposure) were not significant either for the placebo or the MP conditions.

**Figure 4:**
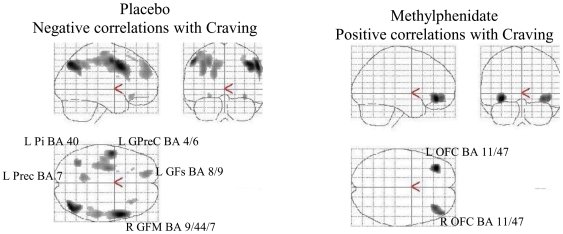
SPM results for the voxel-wise correlation between changes in metabolism and changes in self-reports of cocaine craving (Neutral - Cocaine-cues) for the Placebo and the Methylphenidate conditions (p<0.005 uncorrected, cluster size >200 voxels). For Placebo the correlations were negative (decreases in metabolism associated with increases in craving) whereas with Methylphenidate these were positive (increases in metabolism associated with increases in craving).

In contrast the SPM voxel-wise correlation between changes in metabolism and self-reports of craving identified several regions where these correlations were significant but the pattern differed for placebo and for MP (Figure 4). For the placebo condition increases in craving were associated with decreases in metabolism in left inferior parietal (BA 40), left precuneus (BA 7), left precentral gyrus (BA 4/6), left superior frontal gyrus (BA 8/9) and right medial frontal gyrus (BA 9/44). In contrast the correlations with MP were associated with increases in left and right metabolism in OFC (BA 11/47) for the significance threshold of p<0.005. Lowering the threshold at p<0.05 identified additional positive correlations with striatum, insula and amygdala (Figure S1).

### Effects of covariates (order, age and gender)

**Figure 5:**
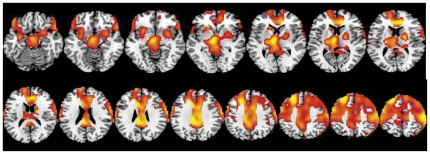
SPM results on comparison (Neutral vs Cocaine-cues video) for the Placebo (PL) and the Methylphenidate (MP) conditions done on normalized metabolic images for the Females (n = 8) and the Male subjects (n = 16). The threshold of significance was set at p<0.005 uncorrected, cluster size >200 voxels. Females showed greater decreases to the Cocaine-cues when compared to the Neutral video than males.

Neither order of the conditions (first, second, third or fourth) nor age had a significant effect. However, covarying for gender showed a significant effect (Figure 5). Females showed a greater reactivity to cues than males.

## Discussion

Here we show in cocaine abusers exposed to Cocaine-cues an unexpected decrease in metabolic activity in limbic brain regions (neutral > cocaine cues) and a blunting of these regional brain responses to Cocaine-cues when oral MP was administered but showed no effect on the subjective experience of craving. Indeed, the cocaine-cues increased craving (self-reports and CCQ) to the same extent with placebo as with MP. This dissociation would suggest that the regions that showed a Cue by Drug interaction effect (regions where responses to the Cocaine-cues were attenuated by MP), are not related to the subjective perception of craving. Indeed the lack of a correlation between the metabolic changes in these regions and the craving responses support this.

When compared with the neutral video, the cocaine-cues video did not change whole brain absolute glucose metabolism, which indicates that when already watching a video the inclusion of cocaine-cues did not further affect the overall glucose utilization by the brain. This contrasts with a prior study in which we reported a 10% increase in whole brain metabolism upon exposure to the same cocaine-cues video but when compared with a baseline condition (subjects resting with their eyes open with no visual or auditory stimulation) [25], which highlights the sensitivity of the activation patterns detected by imaging studies to the comparison state.

In contrast to the lack of an effect on absolute metabolic measures, normalization of metabolism by whole brain, revealed relative decreases in several brain regions with exposure to the cocaine-cues video as compared to the neutral video. These differences were significantly larger when placebo than when MP was given. Specifically during the cocaine-cues video when given with placebo there were relative decreases in limbic regions that included left lateral OFC, left anterior and posterior insula and left nucleus accumbens (NAc), and in the auditory (left BA 22 and left and right BA 42) and visual areas (left lingual gyrus). In contrast when MP was given the only significant differences were decreases in right visual (inferior BA 18) and right auditory (BA 42) regions but no differences in limbic areas.

Thus, the cocaine-cues video when compared with a neutral video led to a relative decrease in activity of limbic brain regions when given with placebo. This is consistent with preclinical studies showing that exposure to cocaine-cues in chronically treated rats decreased glucose metabolism in limbic regions [26]. However, it differs from prior imaging studies that have shown activation of limbic regions with exposure to cocaine-cues. Specifically fMRI studies with exposure to either a script constructed to evoke cocaine craving or a video of cocaine-cues have shown BOLD signal increases in insular cortex as compared to a neutral script or a neutral video [27], [28]. PET studies using videos to evoke craving have also reported cerebral blood flow (CBF) increases in amygdala and anterior cingulate though decreases in striatum [29] whereas others using scripts reported CBF increases in left insula, NAc and amygdala [30], and studies using recall of cocaine imagery supplemented with tactile and visual stimulation with cocaine paraphernalia reported increases in glucose metabolism in left insula and OFC when compared with recall of family genealogy [31]. Moreover, fMRI studies during cocaine intoxication reported that BOLD signal increases in NAc and amygdala correlated with craving [32] and PET studies during intravenous MP administration reported that metabolic increases in OFC and striatum correlated with craving [33]. This would suggest that activation of limbic regions including NAc, insula and OFC but not their deactivation may underlie the subjective awareness of craving. Indeed, while the correlations between craving and the brain regions deactivated by the Cocaine-cues were not significant, the voxel-wise correlation analysis identified for the MP condition, a positive correlation between craving and right and left OFC (P<0.005), insula, amygdala and striatum (p<0.05 supplemental Figure 1), which is consistent with prior findings (27–32). In contrast, with placebo the correlations were negative and included cortical regions involved with executive control (BA 8, BA 9, BA 44) and regions that are part of the posterior default mode network or DMN (precuneus and inferior parietal regions). Thus this would suggest that individuals who experienced the most craving may show impairment in executive function upon exposure to cocaine-cues. Indeed there is evidence of disrupted executive function in cocaine abusers when performing tasks that involved conditioned-cues (34). Enhanced deactivation (decreased metabolism) of the DMN, which is deactivated when attending to external stimuli (35), would be consistent with a greater engagement of the individual's attention by the Cocaine-cues video than the Neutral video.

In interpreting the results from this study it is crucial to recognize that glucose metabolic measures reflect activity over a 30 minute period and thus the limbic deactivation observed with the cocaine-cues video could reflect a longer lasting effect from exposure to conditioned-cues that may have followed an initial short lasting activation. This is plausible since DA cell firing decreases after the expectation of a predicted reward (from a conditioned stimulus) does not occur [36]. In this respect it is of interest that the only other brain imaging study that measured change in brain glucose metabolism with exposure to a cocaine-cues video and that reported increases in prefrontal cortex, amygdala and cerebellum was done in cocaine abusers who were told that at the end of the study they would receive a dose of cocaine [37], which contrasts with our experiment for which cocaine abusers were not expecting to receive cocaine post-session. In fact, studies in the nicotine field have shown that expectation of drug delivery post-session modulates cue-responses [38]. Future studies are required to assess the behavioral significance of the metabolic decrements observed with exposure to conditioned-cues and the consequences of their attenuation by MP. For example, does the impairment in executive function following exposure to cocaine-cues [34] improve with MP?

The discrepancies between our findings of decreases in limbic metabolism and prior imaging studies showing limbic activation with cues exposures are also likely to reflect the sensitivity of brain responses to the mechanisms used to elicit craving (and as importantly the control condition used to compare this) and to the imaging methods used to measure them (fMRI, PET-CBF, PET-metabolism). For example, the much higher temporal resolution of fMRI enables the detection of areas with dynamic patterns of activation/deactivation as has been reported to occur during cocaine-induced craving [32]. Similarly the contrast comparison is also likely to influence the results obtained as discussed above.

The cocaine-cues video when compared with the neutral video decreased relative activity in somatosensory cortices (visual and auditory); specifically BA 42 (auditory integration region) and inferior occipital cortex. Though we do not have an explanation for these decreases we speculate that this may reflect differences in the objects and sounds presented by both videos.

In this study we also show that MP decreased metabolic activity in precentral cortex (BA 6) and posterior cingulate gyrus (BA 30) for the Neutral condition. Since the posterior cingulate is part of the DMN that gets deactivated with stimulation [35] this would be consistent with the interpretation that MP facilitates the deactivation of the DMN. Indeed, in healthy controls we showed that MP (20 mg po) attenuated the brain activation responses to a cognitive task and facilitated the deactivation of the DMN, when compared with a resting condition [39].

The Cocaine-cues significantly increased heart rate and systolic BP and the effect was more robust when MP was given than when placebo was given. This indicates that some of the peripheral responses to conditioned-cues may be amplified by MP. Indeed studies with oral MP in cocaine abusers that were given cocaine showed an enhancement of cocaine's cardiovascular effects [40].

When covarying for gender we showed a significant gender interaction effect; females were more reactive to cocaine-cues than males. This highlights the importance of future studies to separately investigate the effects of gender in cue-reactivity and in responses to addiction treatments. In contrast neither age nor sequence of scans influenced the results.

Here we observed an attenuation of the limbic responses to conditioned-cues by MP that may be beneficial in decreasing the negative effects that exposure to conditioned cues may have on cocaine abusers (i.e., mood, cognitive function, impulsivity). However clinical trials with MP for the treatment of cocaine addiction have been inconclusive [41]. Future studies that evaluate the potential benefits of MP on executive cognitive function following cocaine-cues exposure may help identify potential therapeutic benefits of this medication in cocaine addiction.

### Study Limitations

A limitation for this study is that changes in brain metabolism, which average activity over 30 minutes, may lack the temporal sensitivity to identify the dynamic changes in brain activity elicited by cue exposures as detected with fMRI. On the other hand the metabolic method may help identify the longer lasting consequences that follow exposure to conditioned cues.

Another limitation is the use of conscious awareness of craving as the dependent variable. Indeed it is likely that unconscious responses to conditioned-cues responses are as important in triggering drug consumption. This is particularly relevant since there is evidence that in cocaine abusers there is a disruption between interoceptive processes and subjective awareness [42]. Another limitation was that we did not record eye tracking nor did we require responses during the video exposure that would have assured us that the subjects were attending to the video. However the fact that the subject's heart rate and blood pressure increased while they watched the cocaine-cues video indicates that the subjects were attending to the stimuli.

It would have been desirable to include a control group to assess the specificity of MP effects to conditioned-cues in addicted individuals.

Finally though we hypothesized that oral MP would blunt the responses to conditioned cues by increasing DA tone and blunting phasic DA signals triggered by conditioned cues we can not determine that this was the mechanism responsible for the attenuation of the regional brain metabolic changes triggered by the Cocaine-cues. PET studies to directly measure DA changes triggered by cues with and without oral MP are necessary to corroborate this.

### Summary

These results document a blunting of regional brain metabolic responses to cocaine-cues by oral MP but failed to corroborate our hypothesis that this would be associated with a parallel blunting in craving. The functional significance of the limbic deactivation after exposure to conditioned-cues and its blunting by MP merits further investigation.

## Materials and Methods

### Subjects

Twenty-four active cocaine abusers (16 M and 8 F; 42±6 years of age; 13±2 years of education) who responded to an advertisement were studied. Subjects fulfilled DSM-IV criteria for cocaine dependence and were active users for at least the prior 6 months (free-base or crack) with average cocaine use of 3.5±2 grams/day; 15±5 years of abuse and average time of last cocaine use was 2±2 days. Exclusion criteria included current or past psychiatric disease other than cocaine or nicotine dependence (22 subjects were smokers); past or present history of neurological, cardiovascular or endocrinological disease; history of head trauma with loss of consciousness greater than 30 minutes; and current medical illness. Scores for the “drug” domain of the Addiction Severity Index [43], corresponded to 8.6±2; and scores for the Cocaine Selective Severity Assessment Scale [44] corresponded to 27±5. Written informed consent was obtained from all subjects.

### Behavioral Scales

To assess the subjective experience of craving we used an analog scale (1–10) for self-reports of “cocaine craving” and the brief version of the Cocaine Craving Questionnaire (CCQ) [24], which evaluates current cocaine craving on a seven-point visual analogue scale. These measures were obtained prior to drug administration (placebo or MP), prior to video exposure, and 40 minutes after (post) initiation of the video. Repeated ANOVA was used to compare differences between neutral and cocaine-cues and to assess if there was an interaction effect between video and drug conditions.

### Cardiovascular measures

Heart rate and blood pressure were monitored continuously throughout the procedure from which we computed the measures obtained 40 minutes prior to the video presentation (Pre) and those obtained 40 minutes after initiation of the video (Post). For the statistical comparisons we used the difference between the Pre and the Post measures and expressed these as percent change from the Pre measures. Repeated ANOVA was used to compare differences between neutral and cocaine-cue and to assess if there was an interaction effect between video (neutral vs cocaine-cures) and drug (placebo vs MP).

### Scans

PET scans were conducted with a whole-body, high-resolution positron emission tomograph (Siemens/CTI ECAT HR+, with 4.6×4.6×4.2 mm NEMA (National Electrical Manufacturers Association) using ^18^FDG. Details on the methods for scanning have been published [45]. Briefly, a 20 minutes emission scan was started 35 minutes after injection of 4–6 mCi of ^18^FDG. Arterialized blood sampling was used to measure ^18^F in plasma. All subjects were scanned four different times each on a separate day under the following randomly ordered conditions (1) oral placebo while watching a Neutral video; (2) oral placebo while watching the cocaine-cues video; (3) oral MP (20 mg) while watching the neutral video; and (4) oral MP (20 mg) while watching the cocaine cues-video. The order of the 4 scans was randomly varied so that only two subjects ended up by having the same sequence of conditions.

**Figure 6:**
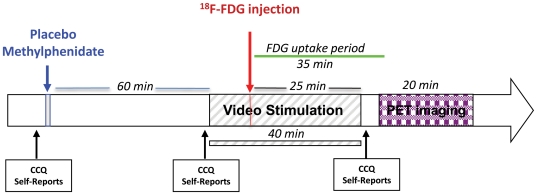
Diagram of experimental protocol.

Videos were started 15 minutes prior to injection of ^18^FDG (60 minutes after placebo or MP administration) and were continued for 25 minutes after ^8^FDG injection (total of 40 minute exposure to the video). Figure 6 shows a diagram of the experimental procedures. The neutral video featured non-repeating segments of nature stories and the cocaine-cue video featured non-repeating segments portraying scenes that simulated purchase, preparation, and smoking of cocaine [14].

### Image and data Analysis

The data were analyzed using a general lineal model with the Statistical Parametric Mapping (SPM) software package [46]. The SPM analyses were performed on metabolic images that were normalized to whole brain metabolism, which increases the sensitivity to regional effects. The images were then spatially normalized using the Montreal Neurological Institute (MNI) template provided in SPM2 and subsequently smoothed with a 16 mm isotropic Gaussian kernel. The four relative metabolic maps corresponding to different conditions (cocaine video, neutral video, placebo, and MP) for each subject were included in a one-way (within-subjects) repeated measures ANOVA model in SPM2. Three variables, session order, age of the subjects and gender were used as covariates in the analyses. Significance was set at p<0.005 (uncorrected, >100 voxels). Statistical maps were overlaid on an MRI structural image.

Pearson product moment correlations were performed to assess the relationship between the difference in activation in brain regions that differed between the Neutral vs the Cocaine-cues video (with placebo or with MP) and the changes in cocaine craving. For this purpose we obtained the metabolic values in circular regions of interest (diameter 7 voxels) placed on the center of the clusters identified by SPM as differing between the Neutral and the Cocaine-cues videos. We also performed voxel-wise correlations between the changes in metabolism (neutral vs Cocaine-cue) and changes in measures of craving and significance was set at p<0.005 (uncorrected >100 voxels).

## Supporting Information

Figure S1 SPM results for the voxel-wise correlation between changes in metabolism and changes in self-reports of cocaine craving (Neutral - Cocaine-cues) for the Methylphenidate condition for a threshold of significance of p<0.05 uncorrected, cluster size >200 voxels.
(2.06 MB TIF)
