# Supplementary figures and images for: Methylphenidate Attenuates Limbic Brain Inhibition after Cocaine-Cues Exposure in Cocaine Abusers
Source: PLoS One. 2010 Jul 9;5(7):e11509. doi: 10.1371/journal.pone.0011509 (PMC2901385; doi:10.1371/journal.pone.0011509)

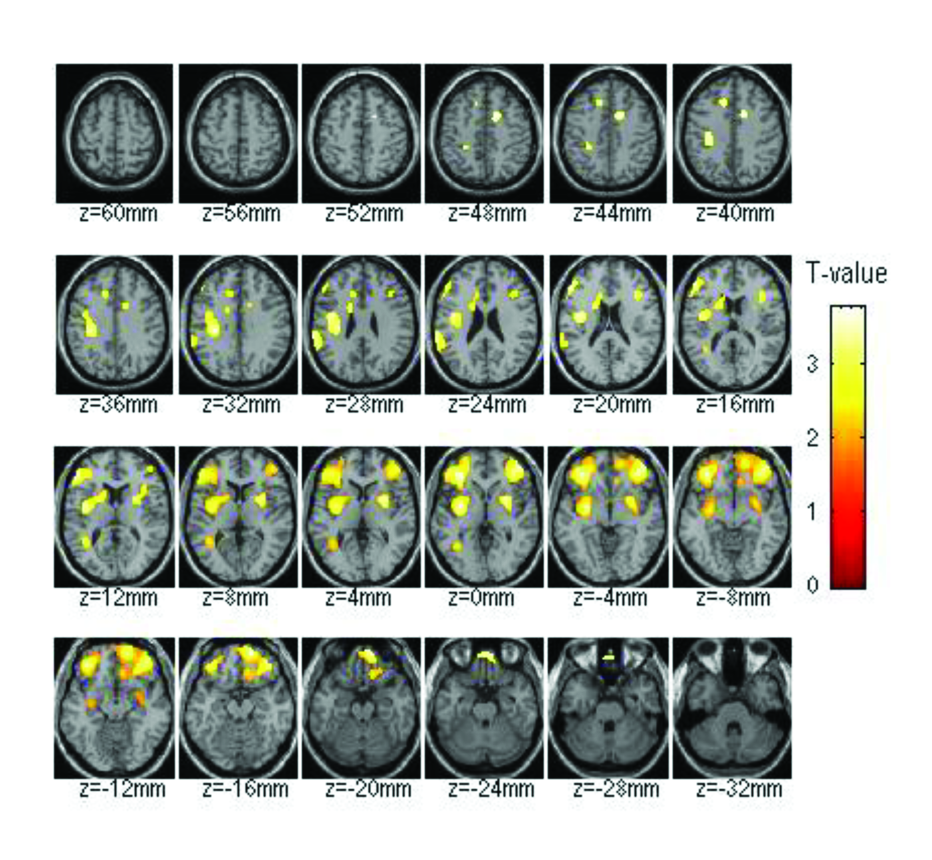

Supplement: Figure S1 — SPM results for the voxel-wise correlation between changes in metabolism and changes in self-reports of cocaine craving (Neutral - Cocaine-cues) for the Methylphenidate condition for a threshold of significance of p<0.05 uncorrected, cluster size >200 voxels. (2.06 MB TIF) [file pone.0011509.s001.tif]
